# A comparison of the efficacy and tolerability of treating primary nocturnal enuresis with Solifenacin Plus Desmopressin, Tolterodine Plus Desmopressin, and Desmopressin alone: a randomized controlled clinical trial

**DOI:** 10.1590/S1677-5538.IBJU.2019.0448

**Published:** 2020-11-18

**Authors:** Parvin Mousavi Ghanavati, Dinyar Khazaeli, Mohammadreza Amjadzadeh

**Affiliations:** 1 Golestan Hospital Tehran Iran Golestan Hospital, Iran, Tehran, Republic of Islamic; 2 Ahvaz Jundishapur University Tehran Iran Ahvaz Jundishapur University, Ahvaz, Khuzestan, Iran, Tehran, Republic of Islamic

**Keywords:** Nocturnal Enuresis, Solifenacin Succinate, desmopressin, valyl(4)-glutaminyl(5)- [Supplementary Concept]

## Abstract

**Introduction:**

Nocturnal enuresis (enuresis) is one of the most common developmental problems of childhood, which has often a familial basis, causes mental and psychological damage to the child and disrupts family solace.

**Objectives:**

In this study, we compared therapeutic efficacy and tolerability of treating primary nocturnal enuresis (PNE) with solifenacin plus desmopressin, tolterodine plus desmopressin, and desmopressin alone. Because we don't have enough information about this comparison especially about solifenacin plus desmopressin.

**Patients and Methods:**

This clinical trial study was performed on 62 patients with enuresis aged 5-15 years who referred to the urology clinic of Imam Khomeini Hospital in Ahwaz in 2017-2018. Patients were randomly assigned to one of the three different therapeutic protocols and any participants were given a specific code. After that, we compared the therapeutic response and the level of satisfaction of each therapeutic group in different months. Data were analyzed using SPSS 22 software and descriptive and analytical statistics.

**Results:**

The mean age of patients was 8.70±66 years. In the therapeutic group with desmopressin and solifenacin, 19 of 20 patients (95%) achieved complete remission (1) after a 3-month treatment in comparison with monotherapy group in which 14 of 22 patients (63.63%) achieved complete remission; and in the combination therapy group of desmopressin and tolterodine, in the study and the evaluation of the consequences of 3-month treatment of this group, it was found that 17 of 20 patients (85%) had complete remission. Overall, the therapeutic response in combination therapy groups of desmopressin plus anticholinergic was higher than the monotherapy group of desmopressin alone.

**Conclusion:**

Our results demonstrate that the combination of desmopressin and an anticholinergic agent is highly effective in treatment of children with PMNE. Although desmopressin has long been a first - line treatment for PMNE, desmopressin monotherapy often fails to achieve a successful response in patients with PMNE.

## INTRODUCTION

Nocturnal enuresis (NE) is one of the most common types of urinary incontinence in children, which has often a familial basis. According to International Children's Continence Society (ICCS), the enuresis is a discrete portion of wetting while asleep in children older than 5 years of age, approximately at the age of 7 years old. There are more outbreaks, however in 2-3% of children it may continue until adulthood (2). Previous studies have demonstrated that 15-20% of 5-year-old children, 5% of 10-year-old children, 1-2% of individuals aged 15 years and 2% of young adults, suffer from NE (3). NE has more prevalence among 8-11 years old boys (4). NE has psycho-emotional effects on child or adolescent such as instigate anger, punishments, rejection in caregivers and loss of self-confidence (5).

The NE has complex, multifactorial pathogenesis and despite numerous studies, its etiology remains elusive. Studies proposed three main factors in the pathophysiology of enuresis including high nocturnal urine production, nocturnal low bladder capacity or increased detrusor activity and arousal disorder (6). Nocturnal enuresis is divided into two categories, primary and secondary. Primary enuresis is considered when the child never had dry bedding for six months and a common cause of this type is a delay in the development and function of the bladder. Secondary nocturnal enuresis is fitted when the child begins to have NE after at least six months of dry nights (7).

The treatment of nocturnal enuresis is classified into pharmacological and non-pharmacological. Non-pharmacological treatments include urotherapy, limitation of fluid intake and bedwetting alarms. The main pharmacological treatments include an arginine vasopressin analog (Desmopressin®), tricyclic antidepressants (Imipramine®), and anticholinergic drugs (tolterodine and solifenacin) (3).

Desmopressin (DDAVP) is a selective vasopressin V2 receptor agonist that connects to the receiver tubes and increases water permeability of collecting ducts so that increases the absorption of water and reduces water leak and disposal of urine in patients with nocturnal enuresis (1, 2). Desmopressin is considered a first-line drug therapy for enuresis. It is approved for treating nocturnal enuresis (8). Lottman et al., evaluated desmopressin treatment in the real-life clinical setting with a large-scale, 6-month investigation of efficacy and safety in patients with severe PNE; as a result, they reported that desmopressin works in about 41% of children with ≥50% reduction in bed wetting nights (9). However, in a recent randomized prospective study, the long-term success of desmopressin and enuretic alarm therapy in children with PNE were investigated. They declared that 77% of those receiving desmopressin achieved more than 90% reduction (10).

Some studies investigated the role of anticholinergics or parasympathetic antagonist's drugs in improving the function of the bladder capacity (11, 12). In monosymptomatic nocturnal enuresis (MNE) children, when the first-line therapy with desmopressin failed, the ICCS recommends combination therapy. In family of anticholinergics, oxybutynin has passed the test of time; because of its side effects, tolterodine with its lower side effects has gained attention in recent literature (13) and recently Solifenacin, the newest member of these drugs family has the longest half-life and its superiority to oxybutynin and tolterodine has been proven for the treatment of overactive bladder in adults (14). In a recent clinical trial by Ravanshad et al., the efficacy of desmopressin and oxybutynin combination therapy in PNE children were assessed. They declared that in the treated group with combination therapy, 83.34% were cured in 1 month and 86.7% in 3 months (15). Furthermore in a study by Azarfar et al., it was revealed that combined treatment with desmopressin plus tolterodine performs better than desmopressin plus oxybutynin in PMNE children (13). Thus, according to previous studies, the objective of this study is to compare the additive efficacy of tolterodine and solifenacin to desmopressin with for the treatment of PMNE.

## MATERIALS AND METHODS

### Study design

This double-blind, controlled trial study was conducted at urology clinic of Imam Khomeini Hospital in Ahwaz from 2017 to 2018. Among patients with PNE, 62 eligible with 5-15 years old after met the inclusion criteria participated in the study. After filling out the informed consent form, patients were randomly assigned to one of the three different therapeutic groups and each participant was given a specific code.

General information regarding age, sex, number of nocturnal enuresis per week were asked and recorded. Among the 62 patients, 22 received desmopressin monotherapy (monotherapy group), 20 received desmopressin and tolterodine medication (Combination therapy group 1) and 22 received desmopressin and solifenacin medication (Combination therapy group 2). Each treatment group received 1 puff of desmopressin nasal spray every night. Also combination therapy group 1 received oral pills of tolterodine with the dosage of 2mg and combination therapy group 2 received oral pills of solifenacin with the dosage of 5mg every night. After receiving the drugs, all patients were evaluated for 3 months and at the end of each month, they were evaluated for their response to treatment and satisfaction, severity of nocturnal and drug complications. The main outcome included the response to treatment in the treated patients.

Medication side effects were assessed by the presence or absence of any clinical complaints that had been reported before.

### The studied side effects of the drugs were as follows:

Desmopressin: seizures (the measurement of sodium level in terms of the risk of hyponatremia), runny nose, nasal congestion and epistaxis, gastrointestinal disturbances such as nausea and stomach cramps and temporary headache and anuria.

Tolterodine side effects: dizziness, weakness, abdominal pain, dysuria, urinary frequency, cough, dry mouth.

Solifenacin side effects: dry mouth, constipation, abdominal pain and nausea.

### Inclusion/Exclusion criteria:

Inclusion criteria consisted of 5 to 15 years old patients, primary nocturnal enuresis, negligible daytime wetting, wet at least 4 times over 4 weeks and normal clinical examination with no neurological or urological cause for the enuresis. Exclusion criteria included age <5 years, secondary enuresis, polysymptomatic, neurologic bladder, neurological disorders, and urinary incontinence disorders.

### Ethical consideration

The research followed the tenets of the last edition of Declaration of Helsinki guidelines, eligible patients provided written informed consent, and the research was approved by the ethical committee.

### Statistical Analysis

After recording the results of observers and the results of the study's data by using descriptive statistical methods, the centrality (mean) and dispersion (standard deviation) were analyzed. To express the degree of agreement between observers, by using 2 × 2 tables and a statistical test of kappa, the kappa values were calculated. To perform statistical tests (t and chi-square), the SPSS software (IBM Crop., Armonk, NY, USA) was used. P <0.05 was considered significant in all cases.

## RESULTS

The studied population included 62 children among which 37 were boys and 25 girls (mean age 8.70±2.66 years; range 5-15 years). There were no statistical differences in age, gender, or baseline weekly frequency of NE between the three groups (Table-1). Among the 62 patients, 22 received desmopressin monotherapy (monotherapy group), 20 received desmopressin - tolterodine medication (Combination therapy group 1) and 20 received desmopressin - solifenacin medication (Combination therapy group 2). Each therapeutic group received 1 puff of desmopressin nasal spray. also combination therapy group 1 received oral pills of tolterodine with the dosage of 2mg and combination therapy group 2 received oral pills of solifenacin with the dosage of 5mg.

**Table 1:** Age, sex and nocturia's repetitions in each group of patients.

		Monotherapy group	Combination therapy group 1	Combination therapy group 2	P-value
Age		7.95±2.90	9.30±2.62	8.95±2.37	0.238
**Sex**					
	Boy	13	11	13	0.810
	Girl	9	9	7	
**Nocturia's repetition**		30.09±12.66	33.10±9.61	40.40±20.92	0.192

At baseline, the patients in the monotherapy group had a mean of 30.09 wet nights per month, but after 1, 2 and 3 months of receiving the monotherapy, the number of wet nights decreased and became 13.41, 12.50 and 12.32 of wet nights respectively.

In the combination therapy group 1, at the beginning of the study, the patients had a mean of 33.10 wet nights per month, but after 1, 2 and 3 months of receiving the combination therapy, the number of wet nights decreased and reached 8.20, 5.55 and 4.55 of wet nights respectively.

**Figure 1:**
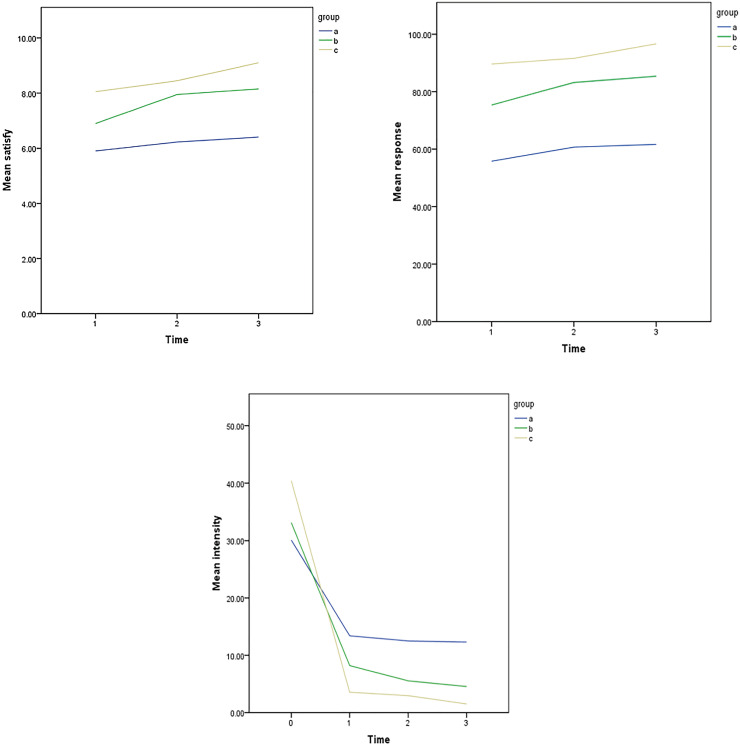
Comparison of the satisfaction, response and intensity of treatment Nocturnal enuresis in a different group (right to left).

In the combination therapy group 2, at first, patients had a mean of 40.40 wet nights in month, but after receiving the combination therapy, the number of wet nights decreased significantly during the monthly follow-ups and it became 3.55 after the first month of treatment, 2.95 after the second month and 1.50 after 3 months of receiving the combination therapy (Figure-1).

According to the response of treatment, among those patients receiving combination therapy of desmopressin and solifenacin, 17 of 20 patients recovered completely after 1-month treatment (85%) and no improvement was observed in 3 patients (15%), Also, after 2-month treatment with this group 18 of 20 patients (90%) achieved a complete remission and no improvement was observed in 2 patients (10%). Furthermore in the study of the consequences of 3-month treatment with this group 19 of 20 patients (95%) achieved a complete remission and no improvement was observed in 1 patient (5%). In comparison, in monotherapy group 12 of 22 patients recovered completely after 1-month treatment (54.54%) and no improvement was observed in 10 patients (45.46s%). In the study of the consequences of 2-month treatment with this group 13 of 22 patients (59.09%) achieved complete remission and no improvement was observed in 9 patients (40.91%). In the study of the consequences of 3-month treatment with this group 14 of 22 patients (63.63%) achieved complete remission and no improvement was observed in 8 patients (36.37%). And in the combination therapy of desmopressin and tolterodine, after 1-month treatment, it was found that 15 of 20 patients (75%) achieved complete remission and 5 patients (25%) were suffering from the same disease. In the evaluation of the consequences of a 2-month treatment with this group, it was found that 16 patients (80%) had complete remission and 20% of individuals still suffered from enuresis. In the evaluation of the consequences of 3-month treatment with this group, it was found that 17 patients (85%) had complete remission and 15% of individuals still suffered from enuresis.

We also assessed the satisfaction level of treatment in all groups after three months of treatment. We used a measurement scale in which the parents chose the satisfaction level of treatment in their patients between the numbers from 0 to 10. We saw that in the monotherapy group, the satisfaction level was 5.91 in assessing the consequences of 1-month treatment. In the study of the consequences of 3-month treatment with this group, the satisfaction level obtained was 6.41.

In the combination therapy group 1, after 1-month treatment, the level of satisfaction was 6.90 and in the evaluation of the consequences of 3-month treatment with this group, this level became 8.15.

In the combination therapy group 2, the satisfaction level in patients was 8.05 in assessing the consequences of 1-month treatment and in study of the consequences of 3-month treatment with this group the level of satisfaction increased and it became 9.10. (Table-2).

**Table 2:** Response, Satisfy and Intensity during the periods of treatment.

		Time	0	1	2	3
	Group					
**Response**	**A**			55.82 ± 30.13	60.70 ± 32.02	61.65 ± 33.02
	**B**			75.39 ± 26.24	83.22 ± 17.47	85.40 ± 17.95
	**C**			89.66 ± 10.94	91.65 ± 9.54	96.66 ± 6.47
**Satisfy**	**A**			5.91 ± 1.77	6.23 ± 2.29	6.41 ± 2.50
	**B**			6.90 ± 1.77	7.95 ± 1.64	8.15 ± 1.81
	**C**			8.05 ± 1.05	8.45 ± 1.64	9.10 ± 1.52
**Intensity**	**A**	30.09 ± 12.66	30.09 ± 12.66	13.41 ± 10.64	12.50 ± 10.87	12.32 ± 11.06
	**B**	33.10 ± 9.61	33.10 ± 9.61	8.20 ± 9.01	5.55 ± 5.68	4.55 ± 5.49
	**C**	40.40 ± 20.92	40.40 ± 20.92	3.5 ± 4.10	2.95 ± 3.72	1.50 ± 3.10

## DISCUSSION

There are different therapeutic approaches for nocturnal enuresis, including behavioral modification, use of the alarm and eventually pharmacotherapy, where in addition to early treatment, pharmacotherapy is also effective in encouraging children to continue behavioral therapy.

In the present controlled trial study, we have accommodated 62 eligible patients 37 boys and 25 girls (mean age 8.70±2.66 years, range 5-15 years) with primary nocturnal enuresis into three treatment groups, treatment with desmopressin alone, combination therapy of desmopressin with tolterodine and combination therapy of desmopressin with solifenacin and we examined therapeutic responses, intensities, satisfactions, and side effects after every month for three months. Overall, the study results revealed that combination therapy of desmopressin with an anticholinergic drug is highly effective in children with PMNE.

Desmopressin monotherapy as the first-line treatment for PMNE often fails to achieve successful response in patients. Previous studies revealed that desmopressin therapy in children has a 60-70% response after 3-month therapy (16, 17). Furthermore, approximately 50% in the desmopressin monotherapy group showed complete responses and also experienced a relapse after the treatment stopped. Therefore, to manage the children who were not responsive to desmopressin therapy, the combination therapy should be considered as the second-line in PMNE treatment (7, 18). In this research, we measured the frequency of nocturnal enuresis in our patients. The initial number of nocturnal enuresis's repetitions (baseline) was 30.09/month in the monotherapy group of desmopressin, 33.01/month in the combination therapy group of desmopressin and tolterodine and 40.40/month in the combination therapy group of desmopressin and solifenacin. We saw that the repetitions rates after three months of treatment respectively decreased to 12.32/month in the monotherapy group of desmopressin, 4.55/month in the combination therapy group of desmopressin and tolterodine and 1.50/month in the combination therapy group of desmopressin and solifenacin. Furthermore, in the third period of treatment, there was a significant difference between the intensities in the monotherapy group and combination therapy group 2. In a study by Park. S et al., the efficacy of desmopressin compared to combination therapy (desmopressin and an anticholinergic) in treatment of PMNE were evaluated, as a result they declared that after 1 and 3 months of combination therapy, the number of wet nights in the treatment group decreased significantly, with a 72.7% and 87.7% improvement in the risk of a wet night, respectively, compared with baseline enuresis frequency. Also compared to baseline enuresis frequency, patients in the monotherapy and combination therapy groups differed significantly in terms of percentage improvement after 1 month and 3 months treatment (19). Moreover, in a randomized controlled clinical trial that studied the effects of primary nocturnal enuresis treatment with oxybutynin plus desmopressin, desmopressin alone or imipramine alone were compared. As a result, it was observed that combined desmopressin and oxybutynin had the best and most rapid results compared to single- drug therapy regimens (20). In another study by Alloussi et al. it was reported that combination treatment showed a significant 66% decrease in the risk of a wet episode after 1 month of treatment compared with the placebo group (17).

In our study, we evaluated the complete recovery rate in study groups. In the desmopressin treatment group, after 1, 2 and 3 months of treatment, respectively, 45.46, 59.09% and 63.63% of patients achieved a complete remission. However, in desmopressin and tolterodine group respectively the complete remission rate increased to 75%, 80%, and 85%. Furthermore, this assessment in desmopressin with solifenacin treatment group showed higher complete remission rates, 85% in 1-month treatment, 90% in 2-month treatment and 95% in 3-month treatment. In all periods of treatment, the response showed an increasing time-dependent manner. Also, the complete remission rate was significantly higher in combination therapy group 2 and lower in monotherapy group in all three times of study. In a randomized controlled clinical trial study, the combined desmopressin and tolterodine efficacy versus desmopressin alone efficacy in the treatment of nocturnal enuresis were evaluated. The results indicated that in a 4-weeks treatment with tolterodine+desmopressin group, 54% of patients had a full response, but in the desmopressin+placebo group, 34% had a full response (21). Our study results are in consistent with Netto et al. studies on better outcomes in combination therapy of PMNE (22). The same in most of the previous studies the combination therapy was given especially to children who were not responsive to desmopressin monotherapy.

Also, we evaluated the satisfaction level of treatment in these three groups. We used a measurement scale in which the parents chose the satisfaction level of treatment in their patients between the numbers from 0 to 10. We found that the patients in the group that were under the combination therapy of desmopressin and solifenacin, had a satisfaction level of 9.1 after three months of treatment. This scale was 6.41 in the group of monotherapy and 8.15 in the group that was under the combination therapy of demopressin and tolterodine. Results revealed that the addition of an anticholinergic agent to the desmopressin regimen will increase the feeling of discouragement and decrease the compliance of the children and their parents. On the other hand, this combination therapy could expose these children to the risk of unnecessary overtreatment. Therefore, clinicians are responsible for choosing the best treatment option for each patient.

The combination therapy in PMNE children is more effective, but its mechanism is not fully understood yet. Overall it seems that success of combination therapy depends on the synergy of desmopressin for decreasing urine volume (23, 24) and anti-cholinergics for increasing bladder capacity, also in managing pediatric enuresis, elevated PVR(post-void residual) is a significant predictor for a lower chance of complete response to treatment (25, 26). After our 3-month treatment period, we observed that the higher responses take place in longer periods maybe due to the fact that time duration and drug synergy affect the responsive or unresponsive treatment outcomes.

## CONCLUSIONS

Nocturnal enuresis (NE) is one of the most common developmental problems of childhood, which has often a familial basis. There are more outbreaks at the age of 7 years old. Our results demonstrate that the combination of desmopressin and anticholinergic agents is highly effective in children with PMNE. Although desmopressin has long been a first-line treatment for PMNE, desmopressin monotherapy often fails to achieve a successful response in patients with PMNE.
